# Waist—What? Can a single sensor positioned at the waist detect parameters of gait at a speed and distance reflective of older adults’ activity?

**DOI:** 10.1371/journal.pone.0286707

**Published:** 2023-06-08

**Authors:** Grainne Vavasour, Oonagh M. Giggins, Matthew W. Flood, Julie Doyle, Emer Doheny, Daniel Kelly

**Affiliations:** 1 NetwellCASALA, Dundalk Institute of Technology, Co. Louth, Dundalk, Ireland; 2 Luxembourg Institute of Health, Strassen, Luxembourg; 3 School of Electrical & Electronic Engineering, University College Dublin, Belfield, Ireland; 4 Faculty of Computing Engineering and The Built Environment, Ulster University, Derry (Londonderry), Northern Ireland; Lunds Universitet, SWEDEN

## Abstract

One of the problems facing an ageing population is functional decline associated with reduced levels of physical activity (PA). Traditionally researcher or clinician input is necessary to capture parameters of gait or PA. Enabling older adults to monitor their activity independently could raise their awareness of their activitiy levels, promote self-care and potentially mitigate the risks associated with ageing. The ankle is accepted as the optimum position for sensor placement to capture parameters of gait however, the waist is proposed as a more accessible body-location for older adults. This study aimed to compare step-count measurements obtained from a single inertial sensor positioned at the ankle and at the waist to that of a criterion measure of step-count, and to compare gait parameters obtained from the sensors positioned at the two different body-locations. Step-count from the waist-mounted inertial sensor was compared with that from the ankle-mounted sensor, and with a criterion measure of direct observation in healthy young and healthy older adults during a three-minute treadmill walk test. Parameters of gait obtained from the sensors at both body-locations were also compared. Results indicated there was a strong positive correlation between step-count measured by both the ankle and waist sensors and the criterion measure, and between ankle and waist sensor step-count, mean step time and mean stride time (r = .802–1.0). There was a moderate correlation between the step time variability measures at the waist and ankle (r = .405). This study demonstrates that a single sensor positioned at the waist is an appropriate method for the capture of important measures of gait and physical activity among older adults.

## 1. Introduction

By 2050 the proportion of the world’s population over the age of 60 years will almost double from 12% to 22% [1]. Biologically, ageing is associated with a gradual accumulation of molecular and cellular damage. This, over time leads to a decline in physiological reserves, functional capacity and an increased susceptibility to disease. In a climate where health services are struggling to meet the needs of older adults [2], mitigating the risks associated with ageing must be a priority of health care providers. This is necessary to reduce the physical and socioeconomic burden associated with functional decline while reducing the demand on health care and long-term care facilities [3]. The majority of older adults wish to remain at home and ageing in place is a key goal of national ageing strategies worldwide [4]. The changes associated with ageing are not consistent among older adults however, and are influenced by extrinsic factors including an individual’s behaviours and the environment [5, 6]. Therefore, shifting the focus from the changes in population distribution to the potential and functional capacity of older adults is perhaps a more constructive and pro-active approach that can alter the impact of an ageing population [7].

According to the World Health Organization (WHO), health is central to our functional capacity, independence and experience of older age [8]. The association between level of physical activity (PA), functional capacity and health is well documented in the literature [9–12] with an increasing body of evidence on the relationship between sedentary behaviours and health. Inactivity is identified as one of the main risk factors for adverse outcomes [8].

Changes in gait parameters are associated with age and have been identified as a key prognostic indicator for disability [13] Parameters of gait commonly measured include speed, step-length, stride-length, time in the double-support phase and variability. Gait-speed has been identified as the strongest predictor of disability, prolonged hospital stay and quality of life [14–16] while variability in gait, specifically step-width variability, has been shown to be negatively associated with levels of PA [17]. Walking is considered the main contributor to PA in adults and as a choice of exercise it increases with age [18]. Therefore, it is important to measure gait parameters as part of assessments of PA. and accurately measuring step-count is an important first step [19]. Measuring PA and identifying those who do not meet the minimum requirements for healthy ageing can increase awareness of inactivity and help target appropriate interventions [20, 21]. PA has traditionally been measured in either self-reported estimates or structured, supervised measurements of gait [22]. Historically objective measurements involved costly, complicated, and time-consuming laboratory-based assessments including force plates, motion analysis capture systems, ECG-based chest straps, direct observation and / or video-recording [23]. Laboratory-based assessments of PA arguably reflect capacity but not necessarily the performance of everyday activity [24]. Self-report measures of PA including questionnaires, diaries or logs, interviews and surveys which provide estimates of PA are by their nature subjective with a risk of bias [25] and have poor validity [22]. Facilitating adults to objectively monitor and quantify their own activity levels and physical function promotes self-care which influences self-regulating and self-efficacy, and facilitates the collection of valuable data over extended periods of time in a real life environment. Both self-monitoring and self-efficacy have been identified as determining factors in exercise behaviours, with higher levels of both associated with better health outcomes [26]. A more proactive approach to self-care and preventative measures can play an important role in supporting healthy ageing and promoting independent living [27].

The proliferation of wearable sensors and their anticipated evolution over the coming years suggests more proactive solutions to healthcare, ageing and functional decline with new developments from the user’s perspective [28]. Their use will enable older adults to take on a more independent, proactive role in monitoring and addressing risk factors thereby developing self-regulation and self-efficacy.

There are many studies to support the use of wearable sensors to detect activity levels among older adults, for example in measuring walking, running, sitting, lying, ascending or descending stairs [29–31]. However, there is little consensus within the literature regarding the best placement of a sensor for accurately and reliably monitoring each different activity [29, 32, 33]. Ankles, thigh and waist sensor placements have demonstrated high accuracy of step detection at different gait-speeds and are therefore useful for different populations [34]. The ankle has been recommended for accurate gait-event detection at slow speeds [35] that may reflect the self-selected pace of older adults. As acceleration signals increase in magnitude from the head to the ankle, highest activity recognition accuracy is anticipated from a sensor positioned at the ankle [30] and as a body-location for sensor-positioning the ankle is the most frequently recommended [36]. However, with ageing comes a reduction in fine motor skills and dexterity which can create barriers to the use of wearable sensors [37]. While the ankle sensor-placement may provide the best quality data it may not be the most accessible location for independent use by older adults. It is therefore important to compare data from different sensor-placements including those more easily accessed by older adults. A study of acceptability and usability of wearable sensors among older adults investigating the preferred body-location of wearable sensors among this cohort found that incorporating a sensor into familiar accessories was the preferred option. Most common areas for placement of accessories included wrist and waist [38]. Wrist-worn activity trackers have shown decreasing accuracy with slower walking speed typical of older adult’s self-selected gait speed [23]. The two body-locations for positioning of the wearable sensors were guided by previous studies [24, 39–44]. The ankle has been suggested as the optimal position for gait detection [18, 45, 46] and is recommended due to its proximity to ground contact point for gait event detection. However, the waist, arguably a more accessible location for use by older adults is a common sensor-location selected in studies [47]. Data obtained from single sensors positioned at the waist have demonstrated high sensitivity and accuracy for falls detection in older adults and percentage error of step-count comparable with that from the ankle at gait speeds reflective of the self-selected gait speed of older adults [46]. Ease of access, acceptability among older adults and wearability have been cited as the reason for waist selection [38].

Identifying a body-location acceptable and accessible to older adults to capture objective measurements of mobility and physical activity will guide further study to examine if older adults can independently capture relevant data over extended periods in a real life setting. This could play a significant role in fostering self-monitoring and self-efficacy thus supporting healthy ageing and promoting independent living [27]. The aim of the study presented in this article was to establish if a single sensor positioned at the waist could capture parameters of gait and PA comparable to that obtained from a sensor positioned at the ankle and to that of a criterion measure of step-count. A research grade sensor was selected to first establish if a single sensor positioned at the waist could provide data with sufficient accuracy before further study to select the most appropriate device for independent use by older adults.

The key contribution of this paper is to compare data obtained from sensors positioned at different body locations and confirm the literature demonstrating that the waist is a suitable body location for a sensor to accurately detect step-count and temporal parameters of gait. It is a first step in identifying the potential for a single wearable sensor to record a single parameter of mobility in older adults that could be used to identify a risk of functional decline. Other studies have included step-count as one of a multitude of parameters to examine frailty [25, 48] but not, to the author’s knowledge as a stand-alone parameter.

## 2. Materials and methods

Participants were recruited through advertisements in a local golf club, tennis club and physiotherapy department. Those interested were assessed for eligibility and fully informed about the study. Two cohorts were recruited, one group aged 18–65 years, and the other > 65 years of age. Inclusion criteria required participants to be healthy, independently mobile, physically capable of performing a series of mobility and physical activity tests, have no cognitive or neurological deficits and have no history in the past 12 months of orthopaedic trauma or surgery. Due to COVID-19 restrictions in place in 2020 [49], a convenience sample of community-dwelling volunteers was recruited. Twenty participants were selected in keeping with similar studies [50–52]. The study protocol received institutional ethics approval (the study was conducted according to the guidelines of the Declaration of Helsinki, and approved by the Ethics Committee of Dundalk Institute of Technology, Ireland on January 07th, 2020) and all participants signed a written informed consent form prior to participation. Participants also fulfilled COVID-19-specific requirements.

### 2.1 Study procedure

**Fig 1:**
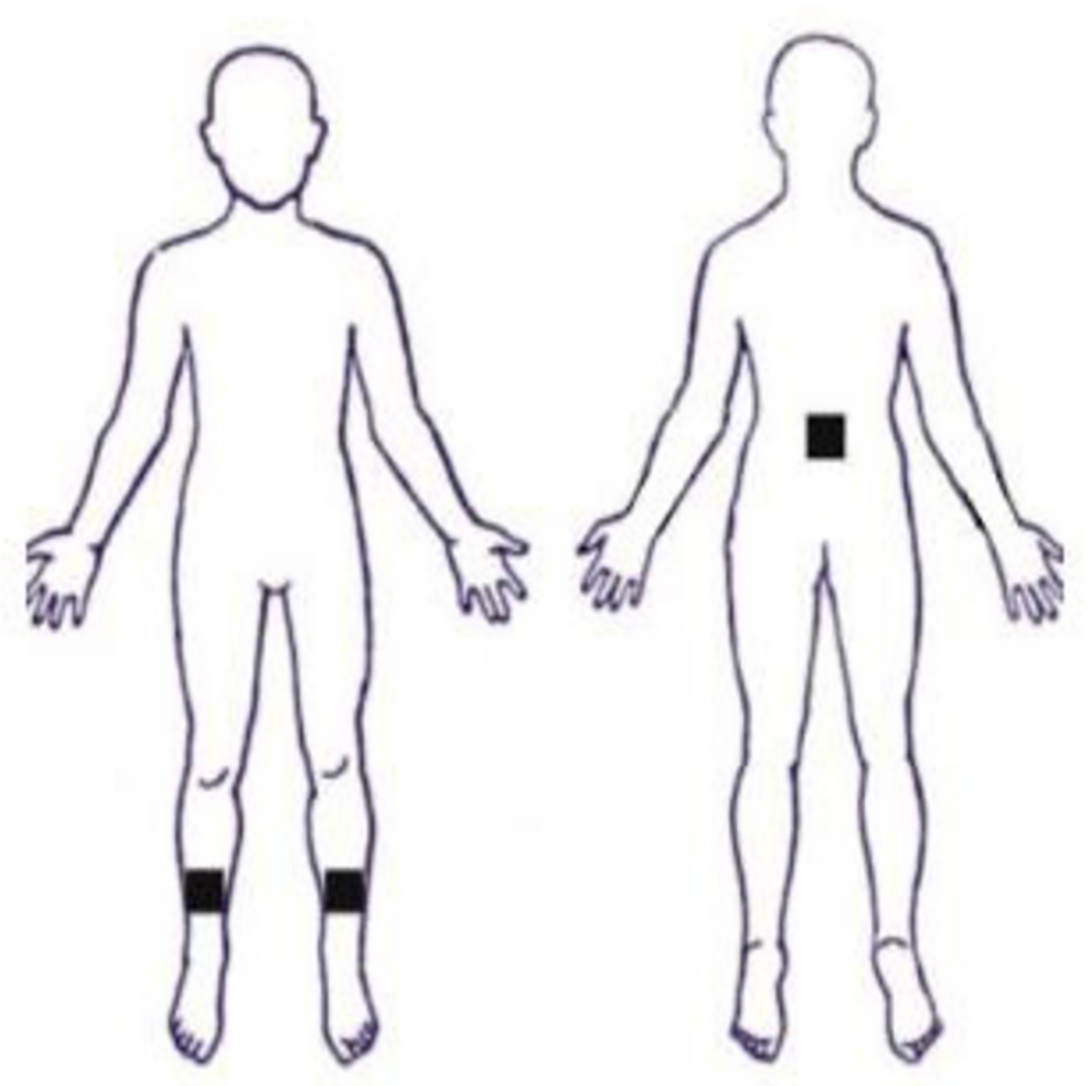
Schematic illustration of sensor placement.

The study was carried out in two different sites for logistical reasons. All participants in the over-65 years of age group were assessed at site one while all those in the 18–65 years of age group were assessed at a separate site (site two). The set-up in both sites were comparable with the exception of the floor surface; a carpet-tile surface at site one and a wooden floor at site two. Measurements of height and weight were taken along with demographic details. Participants were instrumented with inertial sensors (Shimmer Research,) one each placed above the third lumbar vertebra (L3) and bilateral ankles 5cms above lateral malleolus (Fig 1). Each sensor was aligned with the vertical, medio-lateral and anterior-posterior axes of the body and contained a tri-axial accelerometer, tri-axial gyroscope and a magnetometer. Ankle sensors were secured beneath outer clothing, with elastic tubular bandage and tape. The lumbar sensors were secured over outer clothing with a strap and tape.

An inertial measurement unit was selected for use because of its small size, relative low cost and validity and reliability in replacing more traditional, expensive and cumbersome methods of gait analysis [53]. Shimmer inertial sensors have been shown to be accurate in step-count detection and temporal gait analysis in older adults [54, 55].

Prior to data collection, the inertial sensors were calibrated according to the Shimmer 9DoF Calibration procedure [56]. Prior to each participant instrumentation, sensors were configured according to the same application with sampling rate 102.4Hz; acceleration signal range ± 2g; wide range accelerometer, gyroscope and magnetometer sensors enabled; Gyro on the fly calibration and 9DoF algorithms set.

The raw accelerometer data from the sensors were captured and stored onto the on-board memory. This was saved onto a PC on completion of each participant’s sensor data collection for post processing and analysis. Before further processing the accelerometer signal was zero-phase filtered at 50Hz using a 6th order low-pass elliptical filter.

The three-minute treadmill walk test was timed using the treadmill timer (Sole Fitness S77). The three-minute timing started when the treadmill reached the appointed speed (0.8m/s i.e. 2.9km/hr) at approximately 10-seconds. A speed of 0.8 m/s was adopted because it is reflective of the speed of community-dwelling older adults and is a useful cut-off point for prediction of adverse outcomes [57]. Participants were instructed to hold the handrail if preferred, to take long steps toward the front of the moving belt and to keep walking on completion of the three-minutes until the treadmill belt came to a standstill. The three-minute time ended the recording of sensor-steps. The extra steps taken were not included in the manual or the sensor step-count and therefore not analysed. All participants reported being familiar with treadmill use so no period of familiarisation was given. The criterion measurement of steps taken during the treadmill test was determined by a manual step-count performed by direct observation by the researcher in real-time with the 18–65 years of age group only. In the > 65 years of age cohort, video recordings were made during each walk test. Retrospective observation of these video-recordings was used to obtain the criterion measurement of step-count in this > 65 years of age cohort, as it was perceived that this cohort may require more assistance and support during the test, limiting the researcher’s ability to manually count steps in real-time.

### 2.2 Data processing and analysis

To measure temporal features of the gait cycle, accelerometer data from the Shimmer sensors positioned at the waist and both ankles during the treadmill walking test were used to estimate intial contact and final contact times using the Teager-Kaiser gait event detection algorithm (TK_GED_) [58, 59]. The TK_GED_ algorithm first transforms the acceleration signal in the anterioposterior axis using the Teager-Kaiser energy operator and then applies a two-step peak finding method to identify initial and final contact events [62]. Amplitude and temporal threshold scaling factors of the TK_GED_ algorithm were chosen with respect to step time and stride time for data from the waist-mounted and ankle-mounted sensors, respectively. From the estimated initial and final contact times, step-count, mean step/stride time, step time variability, and stride assymetry features were derived for both waist-mounted and ankle-mounted sensors [61]. Step Time Variability was calculated as standard deviation of step times. Stride asymmetry was calculated as the absolute difference between left and right mean stride times. Both are measured in seconds (s). This data processing was performed using MatLab 2020a (MathWorks,).

### 2.3 Statistical analysis

Statistical analysis was performed using Microsoft Excel-16 and SPSS-26 (IBM). Descriptive statistics of continuous variables are presented as Mean and Standard Deviation (SD). Data were tested for normality using the Shapiro-Wilk test. A *p* value of < .05 was considered statistically significant. Because of the small sample size, the relationship between sensor-based step-count and criterion and between data obtained from ankle and waist sensors was analysed using Spearman’s rank correlation coefficient. Conventional values for interpreting a correlation coefficient as very strong (0.9–1.0), strong (0.7–0.89), moderate (0.4–0.69) or weak (0.10–0.39) were used in the analysis [60]. Bland-Altman plots demonstrate limits of agreement with a probability of 95% in each variable for each age cohort. The x-axis of the BAP represents the mean of both measurements using the formula:

criterion+newsensordata/2’

while the y-axis represents the difference between each method using the formula:

criterion−sensordata

[61].

Each age cohort was analysed separately. As a measure of accuracy between the criterion measure of observed step-count and that obtained from the ankle and waist-mounted sensors, and between the gait parameters obtained from the ankle and waist-mounted sensors, mean absolute error (MAE) and mean absolute percentage error (MAPE) were calculated. The MAPE was calculated using the formula *criterion–sensor / criterion x 100* When comparing sensor data from each location the ankle was taken as the criterion. Intra-rater reliability for the observed step-count during the treadmill walk test in the older age group was assessed using the intraclass correlation coefficient (ICC) (2-way mixed-model single measure) with 95% Confidence Intervals (95% CIs). An ICC of >0.80 was considered high, 0.60–0.79 moderate and < 0.60 was considered to be poor relative reliability [62].

## 3. Results

Twenty participants were enrolled in the study; 10 healthy older adults aged > 65 years (age 68.7 ± 3.68, height 164.85 ± 7.45, weight 71.75 ± 11.52, female n = 5) and 10 healthy young adults aged 18–65 years (age 47.7 ± 11.49, height 173.5 ± 8.76, weight 75.5 ± 13.91, female n = 5). Researcher-error regarding timestamps resulted in missing data from n = 2 participants in the >65 years of age group therefore data from eighteen participants were included in the analysis. The results of the Shapiro Wilk test in each age group indicated a significant departure from normal distribution of variables however skewness fell within acceptable range of ± 2 at *p* value <0.05 [63].

**Fig 2:**
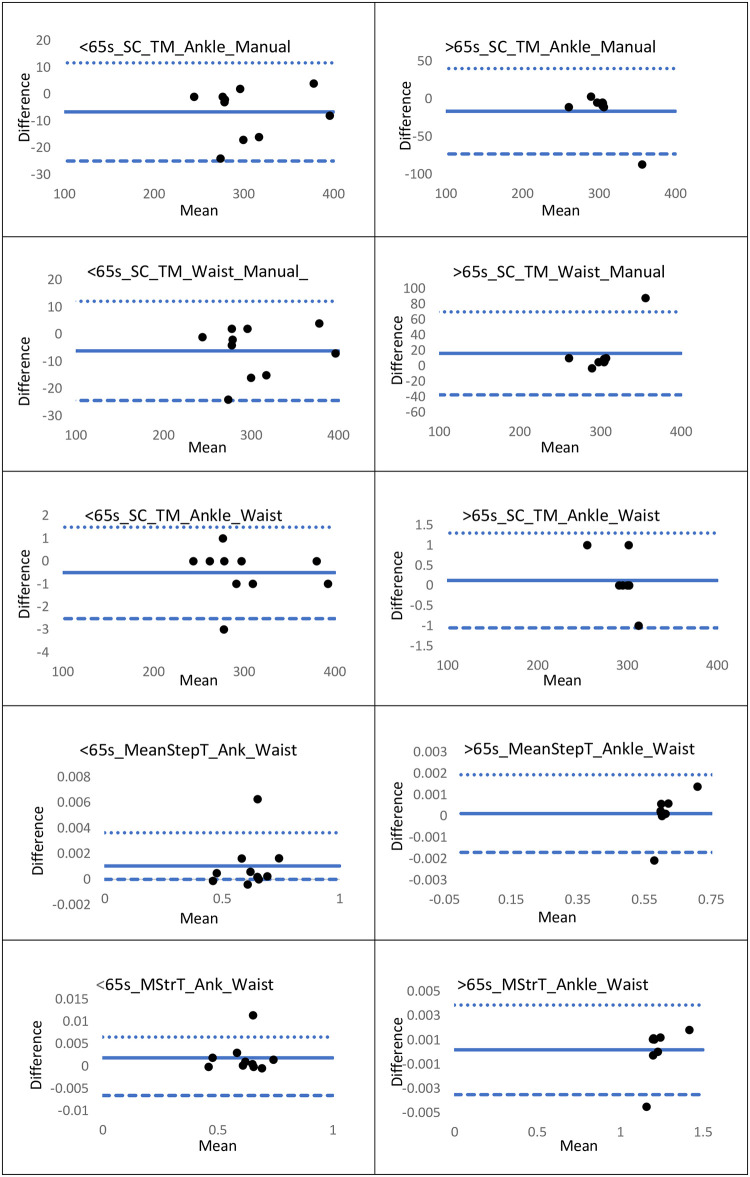
Bland-Altman plots demonstrating level of agreement between; ankle-sensor and manual step-count; waist-sensor and manual step-count; ankle and waist sensor step-count; ankle and waist sensor mean step time; ankle and waist sensor mean stride time. Abbreviation: >65 years of age cohort (>65s). 18–65 years of age cohort (<65s). Manually observed step-count (Manual); Step-count (SC); Mean (M); Time (T). Treadmill (TM). Mean Stride Time (MStrT). ••• Upper Limit of agreement (LOA); **⁃ ⁃ ⁃** Lower LOA; **₋₋₋₋** Bias.

Results of the Spearman’s rank correlation coefficient indicated there was a strong positive correlation between step-count measured by both the ankle and waist Shimmer sensors and the criterion measure in both groups (Table 1). Intra-rater reliability for the criterion step-count as measured in the older age group was excellent (ICC .997, CI’s.986-.999 *p* < .001). Video-recording of the younger age group was not captured and so intra-rater reliability could not be examined for this cohort. There was a strong positive correlation between ankle and waist sensor step-count, mean step-time and mean stride time in both age groups. There was a moderate, not statistically significant correlation between the step-time variability in the >65 years of age group and poor correlation in the younger age group. Stride asymmetry showed poor correlation in both cohorts (Table 2). Bland-Altman plots demonstrating limits of agreement for each group and variable are presented in Fig 2.

**Table 1:** Comparing step-count obtained from ankle and waist-mounted sensors with criterion.

Group	Sensor Location	Sensor Step-count Mean (SD)	Manual Step-count Mean (SD)	Mean Absolute Error	Mean Absolute Percent Error	r_s_
**Age 18–65 years**	Ankle	300.60 (48.53)	307.20 (47.75)	6.6 (9.33)	2.16 (3.08)	.912**
	Waist	301.10 (48.63)		6.1 (9.30)	2.37 (3.09)	.875**
**Age >65 years**	Ankle	294.88 (17.31)	311.25 (38.96)	16.37 (28.8)	4.56 (7.11)	.802*
	Waist	295 (16.89)		16.25 (29.2)	4.50 (7.21)	.878**

Abbreviations:, Spearman’s rank correlation coefficient (r_s_), Standard deviation (SD).

**p<0.01,

*p<0.05

**Table 2:** Comparing gait parameters between inertial sensors positioned at two different body locations.

Group	Parameter	Ankle Sensor Mean (SD)	Waist Sensor Mean (SD)	Mean Absolute Error	Mean Absolute Percentage Error	r_s_
**Age 18–65 years**	Step-count	300.60 (48.53)	301.1 (48.63)	0.7 (0.94)	0.16 (0.38)	.964**
	Mean Step Time (s)	.614 (.088)	.613 (.087)	0.01 x 10–2 (.002)	0.16 (0.29)	.988**
	Mean Stride Time (s)	1.22 (.176)	1.22 (.176)	0.19 x 10^−2^ (.003)	0.15 (0.27)	.988**
	Stride Asymmetry	2.15 x 10^−3^ (2.80 x 10^−3^)	1.20 x 10^−3^ (0.7 x 10^−3^)	0.09 x 10^−2^ (.003)	206 (305)	.176
	Step Time Variability	.07 (.04)	.06 (.02)	0.98 x 10^−2^ (.05)	36.67 (27.8)	.200
**Age >65 years**	Step-count	294.88 (17.31)	295 (16.89)	0.38 (0.51)	0.05 (0.22)	.982**
	Mean Step Time (s)	.62 (.04)	.62 (.039)	0.01 x 10^−2^ (.09 x 10^−2^)	0.01 (0.16)	1.0**
	Mean Stride Time (s)	1.23 (.08)	1.23 (.08)	0.02 x 10^−2^ (.002)	0.01 (0.17)	1.0**
	Stride Asymmetry	0.08 x 10^−2^ (0.07 x 10^−1^)	0.07 x 10^−2^ (0.08 10^−1^)	0.01 x 10^−2^ (.001)	149 (165)	.286
	Step Time Variability	.08 (.03)	.04 (.01)	0.04 (.03)	40 (23.8)	.405

Abbreviations: Spearman’s rank correlation coefficient (r_s_), Standard deviation (SD).

**p<0.01

The difference in the MAPE between step-count obtained from criterion measure of visual count and that from the ankle and waist sensors was less than 5% in each age group (Table 1). The MAE between the gait parameters obtained from each sensor-location in each age group was negligible (Table 2).

## 4. Discussion

Globally an ageing population is presenting challenges associated with functional decline, such as disability, increasing hospital admission with increasing length of stay, dependency and institutionalisation, all resulting in an exponential rise in the cost of health care delivery. Objectives of joint initiatives developed by the European Commission, it’s member states and the WHO to promote healthy ageing include empowering older adults to lead the necessary changes deemed essential to mitigate the risks associated with ageing. These changes include increased awareness, early recognition and timely intervention in the management of functional decline [5, 64]. Advances in availability and usability of wearable sensors and increasing acceptability of technology among older adults provide an opportunity for independent monitoring of mobility and activity behaviours. Independent, objective monitoring of activity behaviours could optimise awareness, autonomy and the prospect of timely intervention of appropriate changes through self-management. Mobility is a vital component of independence and contributes to physical, psychological and social well-being [18, 65]. This in turn reduces the risk of declining function, disability and frailty [66].

This study examined the correlation between step-count obtained from a criterion measure of direct observation with that obtained from a validated algorithm [58, 59] applied to data from accelerometers on the ankle and the waist in a cohort of healthy older adults and young adults. Additionally, gait features obtained from sensors positioned at the ankle and waist were compared. Results suggest a strong relationship between the criterion measure of step-count and step-count obtained from each sensor-location in both cohorts. Strong correlations were also observed between gait parameters of mean step time and mean stride time obtained from both sensor-locations. Spearman’s correlation for step-count between the ankle sensor and criterion, while significant in both cohorts, was less so in the older age group. This is consistent with previous studies which suggest reduced accuracy of sensor-derived measures of physical acitivity in an older age group with reduced gait-speed [20, 50]. In this present investigation, while gait speed was controlled with the use of a treadmill, the difference in cadence between younger and older adults is evident in the mean step-count. The discrepancy in the correlation between the criterion and each sensor-location may be due to reduced accuracy of the gait features extracted from data recorded at the waist compared to the ankle as suggested in earlier studies [30, 45]. One such study acknowledges the difference in accuracy between sensor locations is significant statistically but argues the results are close and therefore the difference is marginal in practical terms [30]. The discrepancy between the observed and sensor step-count may also be due to the accuracy of the observed step-count. Intra-rater reliability in the older cohort was excellent but inter-rater reliability was not established. Video recording was not undertaken in the younger cohort and so reliability could not be evaluated.

Other than sensor location, factors that influence the accuracy of step-count and gait parameters detection include gait speed, distance over which data is collected, the algorithm selection and bandpass filter among others. Gait speed and distance has been shown to influence the accuracy of gait parameter detections with slower speeds and shorter distances resulting in more detection error [67, 68]. The gait speed used in this study was selected to reflect that of older adults, and it is a useful cut-off point for the prediction of adverse outcomes [57], while the distance which was dictated by the duration of continuous walking on the treadmill exceeds that of usual walking patterns of adults which involves short duration of walking bouts [69, 70]. Algorithm selection for gait analysis is known to influence results and an algorithm designed for use with ankle positioned sensors will not necessarily be appropriate for data obtained from sensors positioned elsewhere [18]. As slow gait speed results in low acceleration amplitude and amplitude reduces from the ground up, the sampling frequency and bandpass filter will also influence the accuracy of gait parameter detection. This is demonstrated in a study which applied both a standard and a low frequency extension filter to data resulting in substantially different percentage agreements (69% and 97% respectively for hip positioned sensors) [69]. Amplitude and temporal threshold scaling factors of the TKGED algorithm were selected with respect to data from the waist and ankle-mounted sensors.

Within each cohort, the MAE between the criterion measure of step-count and step-count obtained from each sensor location is similar suggesting the accuracy of step-count extracted from accelerometers positioned at the ankle and at the waist are comparable. The MAE values are small (Table 1) suggesting that in both cohorts, each sensor-location is acceptable in terms of accuracy of step-count. This is supported by an earlier study which found negligble differences in accuracy of measured step-count between ankle and waist-mounted sensors during free-living walking [45]. Similarily, a study examining the accuracy of waist and ankle-mounted sensors in gait analysis at speeds reflective of older adults found less than ten percent error between ankle sensor and waist sensor derived step-count [46]. The MAPE between ankle and waist sensor derived step-count in the current study of younger and older adults was 1.6% and 5% respectively. A study comparing the accuracy of ankle and hip-worn sensors (sensor positioned above the iliac crest), in measuring step-count in a group of 30 healthy young adults found the ankle location was more accurate at gait speed between 0.6–1.0m/s, with a mean percentage agreement of 96% compared with that of just 69% at the hip [69]. Percentage agreement increased to 97% for both sensor positions when a low frequency extension filter was applied to increase the sensitivity of the accelrometer signal at low intensity movements.

Temporal parameters of stride asymmetry and step time variability could not be accurately derived from the waist-mounted sensor as illustrated by high MAPE values in both age groups. Video-recording of the treadmill walk test included footage of the lower limbs only, and whether or not a participant held the handrail of the treadmill was not recorded in real-time during the test and so the effect of arm swing on lower limb variability was not considered. However, temporal parameters of mean step time and stride time were consistent between ankle and waist-mounted sensors with MAPE values less than 2% (Table 2). This is consistent with previous studies suggesting high accuracy of waist-mounted sensors for other applications such as fall detection [71]. The reported results support previous research and demonstrate the waist is an appropriate body-location for positioning of a single sensor to capture parameters of gait and PA among older adults. This outcome will contribute to future examination of free-living walking and temporal parameters of gait in older adults as waist-mounted sensors are potentially more suitable for application by older adults than ankle-mounted sensors [38] and may be more suitable for unsupervised monitoring outside of clinical settings [72].

In this study, accelerometer data were captured using research grade inertial sensors. The raw data from these Shimmer sensors requires processing to extract parameters of gait using a gait event detection algorithm. While the results of this study demonstrate that the research-grade wearable sensors used can produce accurate data, this work also highlights the potential difficulties for both clinicians and their clients/patients in monitoring parameters of gait and PA using research grade devices. These barriers include the need for specific algorithms, specialised data extraction and analysis—all of which complicates extracting useful, actionable information from them. The reliance to date on researchers within a specific field of expertise for the analysis of data is acknowledged in the literature [73]. This demonstrates the need for further research to establish if parameters of mobility and PA could be captured using an alternative, commercial, less research-based sensor system that can be monitored and interpreted by older adults, their family members / carers and/or GP.

## 5. Limitations

Limitations to this study include the small sample size, the method of data collection and the securing of the sensors above outer clothing as opposed to adhering directly to the skin. Each is discussed separately in this section. A small sample was selected for convenience during the COVID-19 pandemic in 2020 when travel and contact restrictions remained in place but the sample size is supported in the literature. Data were collected in two different laboratory settings during a structured treadmill walk test. While not unique for data collection to occur in different settings [74] in the interests of consistency the one location for data collection is preferable. It has been suggested that treadmill walking may not reflect real-life walking patterns [45] and that laboratory-based gait analysis demonstrates less variabilty and higher cadence than free-living assessment thus reflecting participant’s “best performance” [70]. However, treadmills have been widely used in clinical studies as they allow for collection of data at controlled speeds. The three-minute time-frame appointed in this study allowed for the capture and analysis of many gait cycles, examination of gait patterns of older adults and examination of any differences in the gait parameters between the two age groups. This work will contribute to future studies examining gait parameters in older adults in free-living conditions.

To accurately determine gait parameters, accurate detection of initial foot contact and final foot contact is necessary [45]. Positioning of the sensors above outer clothing and not directly to the skin may have affected identification of the final contact point of the foot and thus influenced the results of the variability and asymmetry variables. This method was chosen for convenience and with reference to earlier studies [30, 75]. Whether or not a participant held the handrail was not recorded and so the effect of arm swing on lower limb variability was not considered. Future research should ensure sensors are affixed directly and securely to the skin to optimise integrity of the data collected and that all relevant detail is recorded.

## 6. Conclusions

As people age there is a tendency to move less. Having an objective method for older adults to measure their mobility and thus be alerted to any decline in PA may facilitate early intervention and reduce the associated risks. This study demonstrated a strong relationship between the criterion measure of step-count and that obtained from both ankle and waist-mounted sensors in a group of healthy older and healthy young adults. Strong correlations were also observed between gait parameters obtained from both sensor-locations. These results suggest that a waist-mounted sensor provides accurate measures of gait in a laboratory-based study. It is a first step in identifying the potential for a wearable sensor positioned on a body location conveniently accessible for older adults use, to record a single parameter of mobility in older adults that can indicate a risk of functional decline. While it is a limitation that the study was consucted in a laboratory setting, the walking trials were conducted at a walking speed and distance reflective of older adult free-living walking. These results will inform future research into the possible use of waist-mounted sensors for free-living gait analysis in community-dwelling older adults.
